# Unexpected finding of arc of Buhler with celiac artery stenosis during workup for a suspected pancreatic lesion

**DOI:** 10.1093/jscr/rjad178

**Published:** 2023-03-28

**Authors:** Maria Padar, Amie Rieseberg, Sujith Ratnayake

**Affiliations:** Surgical Department, Caboolture Hospital, Caboolture, QLD, Australia; Radiology Department, Caboolture Hospital, Caboolture, QLD, Australia; Surgical Department, Caboolture Hospital, Caboolture, QLD, Australia

## Abstract

Knowledge about normal and pathological anatomical variants is a key point for all surgeons to perform safe procedures and manage unexpected findings. One example of this is vascular anomalies involving the celiac and superior mesenteric arteries (CA and SMA) and their anastomoses. During a routine workup of a suspected calcified pancreatic lesion, an asymptomatic arc of Buhler was found, connecting the CA and SMA, with 90% stenosis of the celiac trunk. This embryological variant, despite being a rare occurrence, has significance in various surgical procedures, including pancreatoduodenectomy, liver transplantation and interventional radiological procedures, such as gastroduodenal artery ligation and embolisation.

## INTRODUCTION

**Figure 1:**
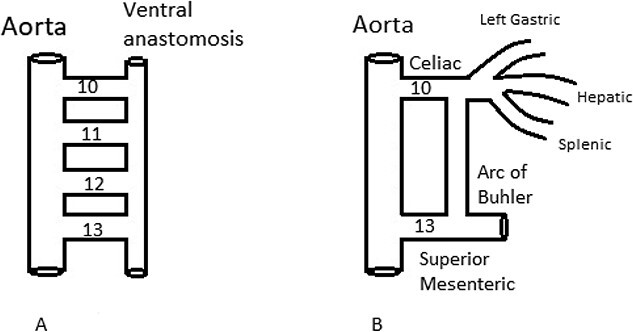
(**A**) Vitelline arteries 10th–13th communication between the aorta and the ventral anastomotic artery. (**B**) The arc of Buhler, embryological remnant of the ventral anastomosis, connecting the CA and SMA, despite the regression of the 11th and 12th vitelline arteries.

The arc of Buhler is an embryological remnant of the ventral anastomosis, connecting the otherwise separated celiac and superior mesenteric arteries (CA and SMA) despite the regression of the 11th and 12th vitelline arteries. During embryogenesis, there are four omphalomesenteric arteries, 10th–13th, allowing communication between the aorta and the primitive ventral anastomotic artery. This is because of the initial dual blood supply to the abdominal viscera via dorsal and ventral arteries. During normal development, the CA arises from the 10th, the SMA from the 13th vitelline artery, whereas the 11th and 12th regress [1, 2] (Fig. 1).

The CA, together with the SMA and the inferior mesenteric artery, is the major blood supply to the abdominal viscera. There are well-known anastomoses between these major arteries. The two main connections between the CA and the SMA are the gastroduodenal and a dorsal pancreatic artery. In rare cases, such as described below, there is a third connection, the Buhler’s arc [3].

## CASE REPORT

A 77-year-old female presented with sudden onset of pleuritic chest pain. Initial investigations showed no ECG changes, normal chest X-ray and normal troponin level. Blood tests were normal. D-Dimer was elevated. Her background history included gastro-oesophageal reflux disease, asthma, chronic kidney disease, polymyalgia rheumatica and anxiety. She had a previous non-provoked PE and completed 6 months of treatment with Rivaroxaban. Her previous thrombophilia screen was negative. CT pulmonary angiogram showed multiple bilateral segmental and subsegmental pulmonary emboli with associated lingular haemorrhage. She was initially treated with Heparin infusion, which was changed to Rivaroxaban. As this episode was her second unprovoked PE without any identified precipitating factors, including immobility, recent major surgery or long-haul travel, it was decided to investigate the possibility of underlying malignant processes.

**Figure 2:**
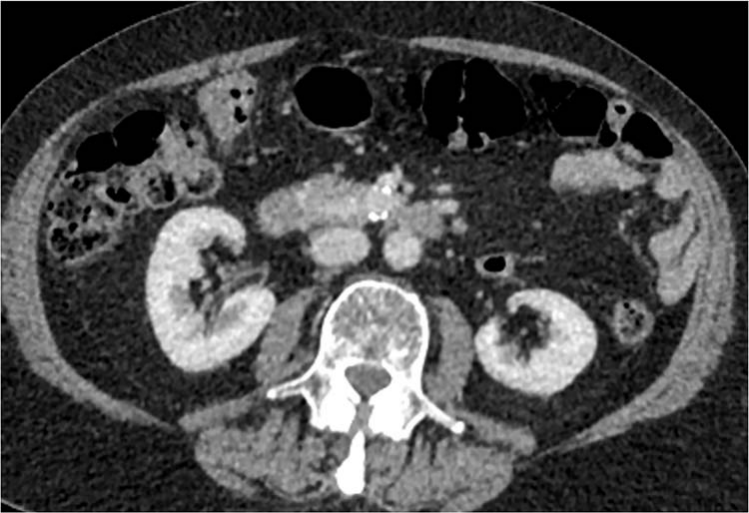
Initial CT: axial view, demonstrating what was initially thought to represent indeterminate pancreatic/duodenal lesion.

**Figure 3:**
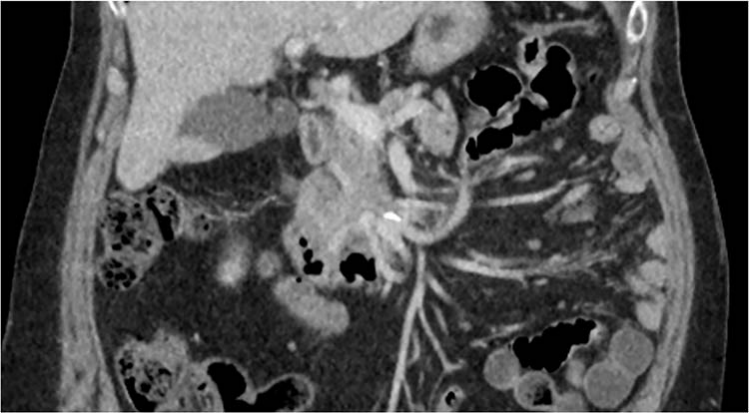
Initial CT: coronal view of a 13 mm hyperattenuating region with peripheral calcification at the inferior margin of the pancreatic uncinate process and the superior margin of the duodenum.

**Figure 4:**
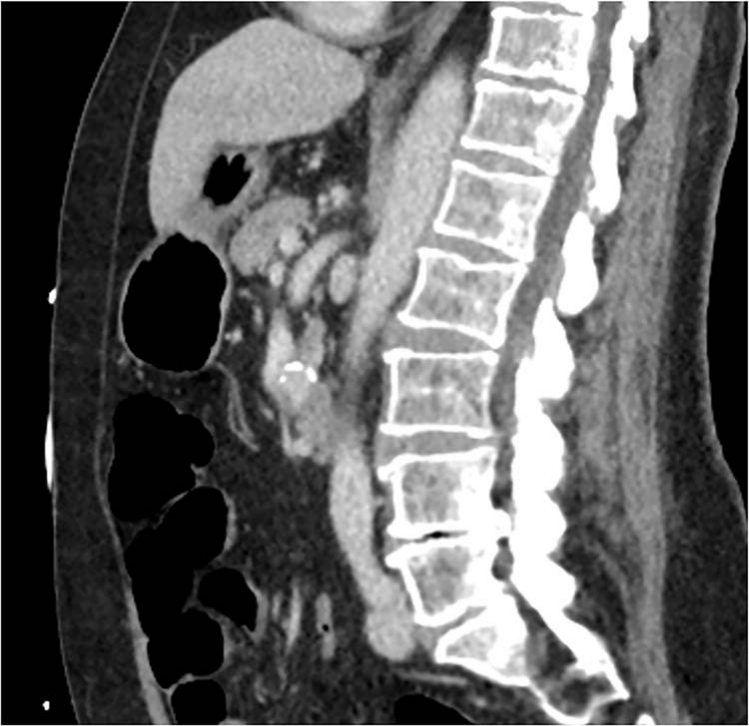
Initial CT: sagittal view of the indeterminate pancreatic/duodenal lesion.

CT of abdomen and pelvis was performed, which revealed a 13 mm indeterminate lesion arising between the pancreatic uncinate process and the duodenum with coarse peripheral calcification, suspicious of primary pancreatic neoplasm or primary small bowel cancer (Figs 2–4).

All specific tumour markers were normal.

She has not had recent abdominal pain, jaundice or weight loss and she had a good appetite. She reported no changes in her bowel habits. There was no history of pancreatitis, or a family history of pancreatic or other malignancies. Ex-smoker with 25 pack year history having quit 38 years ago and social drinker. She had a laparotomy for perforated appendicitis 50 years ago.

**Figure 5:**
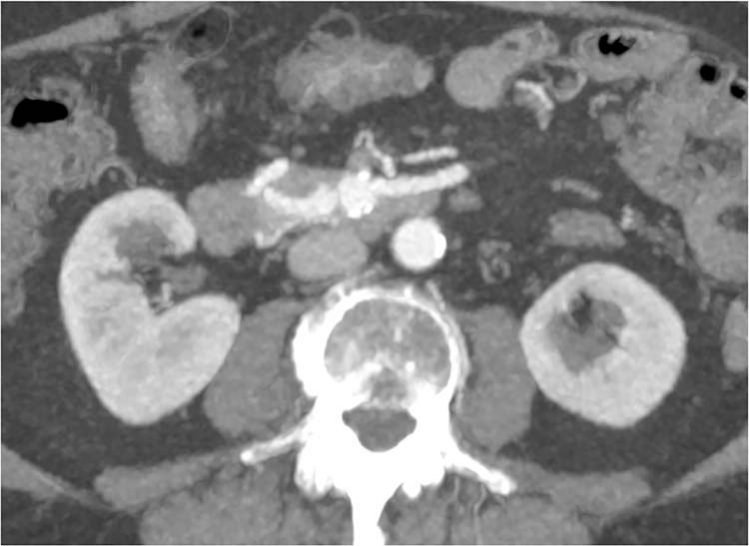
Multiphase CT pancreas, axial view demonstrating abnormal communication between the CA and the SMA.

**Figure 6:**
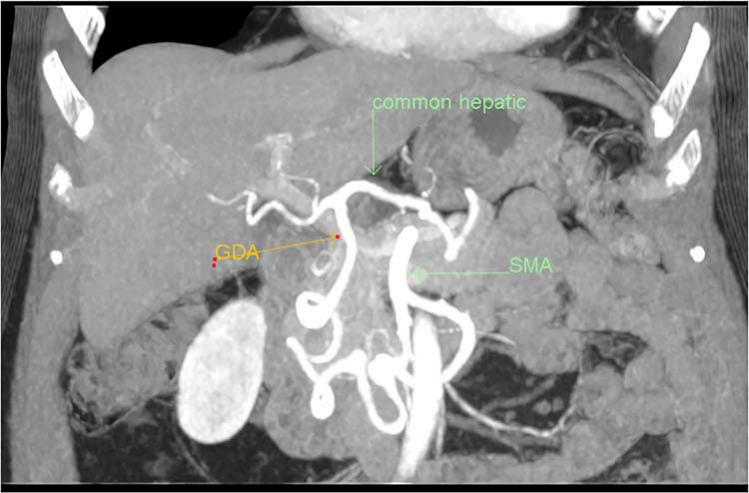
Multiphase CT pancreas, coronal view: the gastroduodenal artery, off the common hepatic, communicating with the first branch of the SMA.

**Figure 7:**
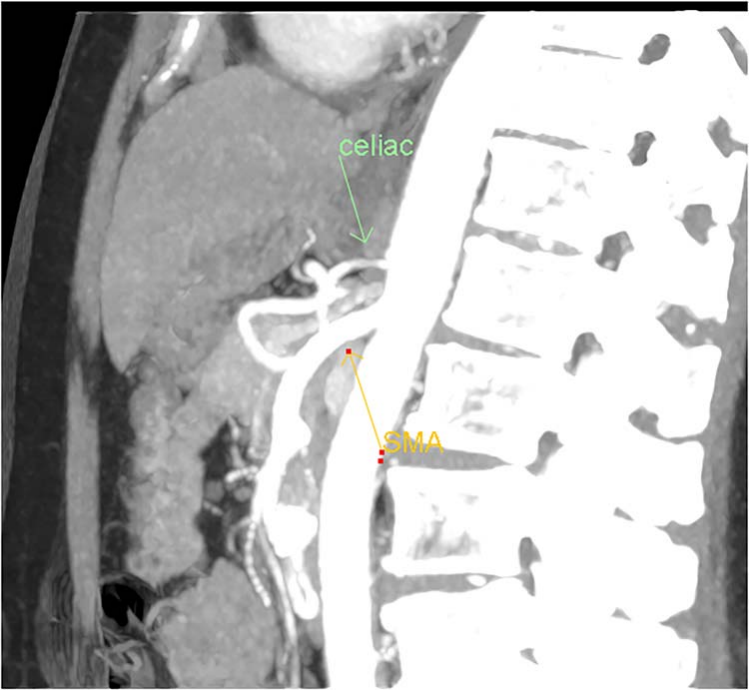
Multiphase CT pancreas, sagittal view: abnormal communication between the CA and the SMA. The celiac axis is markedly narrowed, >90%.

**Figure 8:**
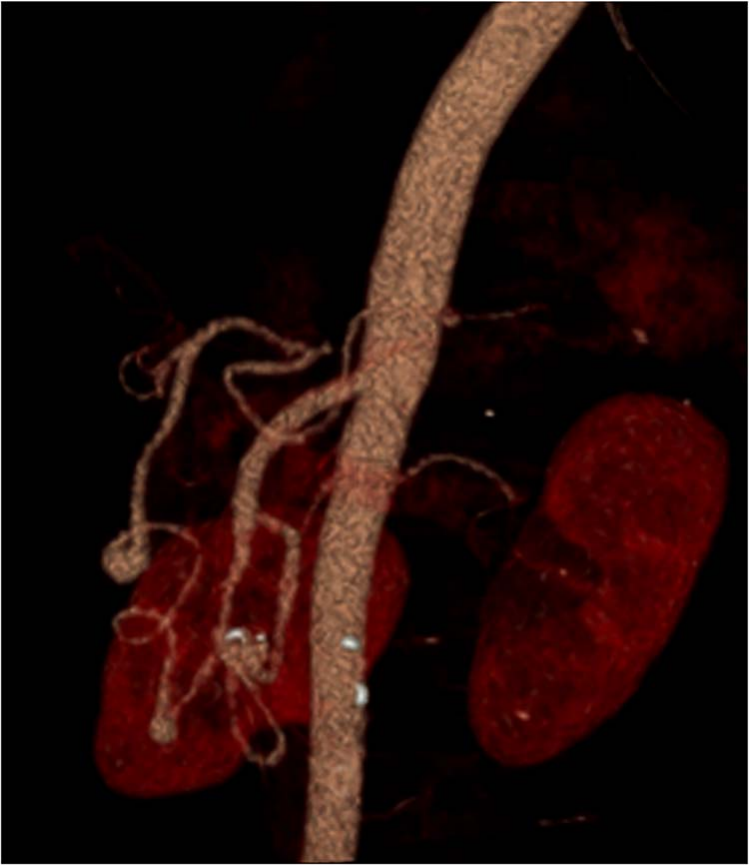
3D reconstruction demonstrating abnormal communication between the CA and SMA.

A multiphase CT of the pancreas had unexpected findings. There were no pancreatic masses on the arterial and portal phase study, and no paraaortic or mesenteric lymphadenopathy. The abnormal calcification seen on the previous study corresponded to a dilated vessel along the superior margin of the pancreatic body. This dilated vessel was an abnormal communication between a tortuous ecstatic gastroduodenal artery and the first branch of the SMA, a Buhler’s arc communication. The celiac axis origin was narrowed >90%. No other suspicious solid organ mass lesion was identified. Soft tissues were unremarkable (Figs 5–8).

The patient was discharged on day three post-admission and currently waiting for a vascular surgical review.

## DISCUSSION

The rare, third anastomosis between the CA and SMA named after Buhler who first described it in 1904 [3]. The theory of linear regression during embryological development of the arc of Buhler was attributed to Tandler [4]. Its incidence varied between 1 and 4% in different studies: <4% in Dubel *et al*. [5], 1.7% in Ferrari *et al*. [6], 3.3% of 120 potential live liver donor patients in Saad *et al*. [7] and 1.0% from 300 angiograms in McNulty *et al*. [8].

Most of the cases were incidental findings on appropriate images of asymptomatic patients, such as our case. There are case reports of ruptured aneurysm of the arc of Buhler causing median arcuate ligament syndrome, fatal retroperitoneal bleed or obstructive jaundice. There is an important association of the existence of the Buhler’s arc in planning of pancreatoduodenectomy in a case of CA stenosis, especially with ligation of the gastroduodenal artery [9, 10].

Awareness of this anatomical variation is also crucial in interventional radiological procedures, such as gastroduodenal artery ligation and embolization of various aneurysms of the gastrointestinal vascular system.

In our case, it was an unexpected, incidental finding of the arc of Buhler during a routine workup for a calcified pancreatic lesion. The patient was asymptomatic, despite a 90% stenosis of the celiac origin because of the collateral blood supply from the SMA via the arc of Buhler.

This case highlights the importance of choosing the appropriate imaging for a pancreatic lesion workup, especially if biopsy is being considered. Additionally, it emphasises the significance of the knowledge of embryological development and vascular variations of the mesenteric blood supply.

## CONFLICT OF INTEREST STATEMENT

None declared.

## FUNDING

None.

## DATA AVAILABILITY

All data and materials can be found in the article. No other data or materials were used.

## INFORMED CONSENT

Informed consent was obtained from the patient for publication of this case report and accompanying figures.
